# Serum Presepsin Might Not Detect Periprosthetic Joint Infection After Hip Arthroplasty

**DOI:** 10.3390/jcm14124246

**Published:** 2025-06-14

**Authors:** Kohei Hashimoto, Takkan Morishima, Kazutaka Watanabe, Tatsunori Ikemoto, Yukio Nakamura, Nobunori Takahashi

**Affiliations:** 1 Department of Orthopedic Surgery, Aichi Medical University Hospital, Aichi Medical University, Nagakute 480-1195, Aichi, Japan; skikohei4145@gmail.com (K.H.); kz0020@yahoo.co.jp (K.W.); 869@mail.aichi-med-u.ac.jp (T.I.); ntakahashi0617@aichi-med-u.ac.jp (N.T.); 2 Department of Orthopedic Surgery, Division of Osteoporosis, Locomotive Syndrome, Joint Disease Center, Aichi Medical University Hospital, Aichi Medical University, Nagakute 480-1195, Aichi, Japan

**Keywords:** arthroplasty, biomarkers, periprosthetic joint infection, presepsin, replacement

## Abstract

**Background**: The purpose of this study was to determine the normative perioperative plasmatic levels of presepsin in patients undergoing primary total hip arthroplasty (THA), and to evaluate whether presepsin measurements can effectively distinguish the presence of periprosthetic joint infection (PJI) following THA. **Methods**: In study 1, we evaluated multiple inflammatory markers before and at several time points after surgery in 31 primary THA patients. The Kruskal–Wallis test was used to compare sequential changes in each variable followed by the Sheffe post hoc comparison. In study 2, we evaluated the diagnostic accuracy of the inflammatory markers for PJI using five cases with confirmed PJI without bacteremia. ROC curve analysis was performed comparing these PJI cases with the 31 preoperative cases from study 1. **Results**: In study 1, presepsin levels were not significantly different from the baseline throughout the monitoring period. In study 2, the AUCs of CRP (1.0, *p* < 0.001) and ESR-1h (0.83, *p* < 0.05) in the ROC curve were able to discriminate PJI, but those of presepsin (0.51, *p* = 0.96) and WBC (0.65, *p* = 0.28) failed to discriminate PJI. **Conclusions**: Our findings suggest that presepsin levels remain stable following THA and may have limited utility in detecting periprosthetic joint infection, particularly in the absence of systemic infection.

## 1. Introduction

Periprosthetic joint infection (PJI) is a serious complication after total joint arthroplasty, requiring an extensive treatment time, significant invasive procedures, and substantial medical costs. Hence it is important to diagnose it and start treatment as soon as possible. Various diagnostic approaches for PJI, including serologic markers, synovial fluid analysis, and microbiological cultures, have been investigated [1,2].

The Musculoskeletal Infection Society’s diagnostic criteria for PJI initially included only C-reactive protein (CRP) and erythrocyte sedimentation rate (ESR) as peripheral blood markers [3]. In 2018, D-dimer was subsequently added as a minor criterion in the updated guidelines [3]. When detecting infection in the body, CRP and ESR are highly sensitive and regarded as useful screening tests, but they carry the disadvantage of low specificity. In fact, Spangeh et al. prospectively investigated whether CRP or/and ESR could be useful for PJI diagnosis at the site of a previous arthroplasty in a 202-case series of revision THAs. They reported that elevations in CRP and/or ESR were often observed in many inflammatory conditions without infection, and thus were regarded as a false positive marker, although the combination of a normal ESR and CRP level could predict the absence of infection [4]. These limitations of conventional inflammatory markers have prompted the investigation of novel biomarkers with a potentially greater specificity for the diagnosis of infection, including presepsin.

In 2005, Yaegashi et al. reported a new biomarker, named soluble CD14 subtype (sCD14-ST) or presepsin [5]. Presepsin (sCD14-ST) is a 13 kDa protein that is a truncated N-terminal fragment of CD14, a known inflammatory marker, which is advantageous in the early diagnosis of sepsis [6]. The secretion of presepsin has been reported as a stimulus of monocyte phagocytosis [7,8], and the range of normal presepsin levels among healthy individuals has been reported to be highly variable [9]. Moreover, the presepsin level cut-off value for discriminating between bacterial and nonbacterial systemic inflammation differs for each report; 600 ng/L [10] and 670 ng/L or 864 ng/L [11].

A previous study demonstrated that presepsin levels increased at 2 hours (h) after the onset of infection, reached a peak at 3 h, and gradually decreased until 8 h in a puncture sepsis model [12]. This biological characteristic has led to presepsin being recognized as a potential early marker of infection.

Recent studies have begun to investigate the feasibility of serum presepsin as a screening marker for periprosthetic joint infection (PJI). In a prospective multicenter study, reference [13] suggested the utility of presepsin values in differentiating between aseptic loosening and PJI. Similarly, Vicenti et al. [14] evaluated perioperative presepsin levels in patients undergoing total hip or knee replacement and reported its potential for detecting postoperative infectious complications. Furthermore, in a prospective observational study [15], established the temporal pattern of presepsin values following cementless total hip arthroplasty and reported that the absence of a decrease at 96 h postoperatively might suggest infection. However, contrasting evidence exists in the literature. In a prospective study, Busch et al. [13] evaluated the diagnostic accuracy of synovial presepsin for PJI diagnosis and concluded that presepsin lacked sufficient diagnostic utility compared to other biomarkers. Their findings directly challenge earlier positive reports and raise important questions about presepsin’s clinical value in PJI detection. These conflicting results highlight the uncertainty surrounding presepsin’s diagnostic utility, and its usefulness for PJI remains insufficiently elucidated. Therefore, we focused our research on presepsin to determine whether it is useful as a serum marker for PJI diagnosis. The purpose of this study was to investigate (1) the normative perioperative plasmatic levels of presepsin in patients undergoing primary total hip arthroplasty (THA) and (2) whether presepsin levels can discriminate the presence of local bacterial infection after THA.

## 2. Material and Method

This study consisted of two series. One was an investigation into the normal chronological course of presepsin levels in patients undergoing primary THA; the other was an investigation into presepsin levels in patients with suspected PJI. This study was approved by the local ethics committee (2015-H182), date 25 January 2018, in our institution, and for both study groups, we employed an opt-out consent methodology in accordance with our institutional ethics committee guidelines. Patients were provided with information about the study and the option to withdraw, with no withdrawals recorded (Table 1 and Table 2).

### 2.1. Study 1

Between May and September 2015, we performed 38 consecutive primary THAs on 36 patients. Cases were excluded if they underwent simultaneous surgeries on both sides, were diagnosed with rheumatoid arthritis, had lost renal function, or were diagnosed with PJI during the course of treatment. This left 31 THAs in 29 patients. In all cases, perioperative antibiotics were routinely administered up to 24 h after surgery and every 6 h starting at 1 h before surgery. All operations were performed using the posterolateral approach. Hybrid THA was performed in 11 hips, and cemented THA was performed in 20 hips. Either coauthor K.W. or T.M. performed the THAs in all patients. All of these cases were confirmed to have been neither suspected of nor diagnosed with PJI at least until the 4-year follow-up after surgery.

### 2.2. Measurement Protocol

Presepsin, white blood cells (WBCs), CRP, and the erythrocyte sedimentation rate 1 h value (ESR-1h) were measured on the day before surgery and on days 1, 3, 5, 7, and 14 postoperatively using the same peripheral blood sample. Regarding the sample collections after surgery, all blood samples were collected in the early morning. Peripheral blood samples were collected by vein puncture and analyzed within 2 h. WBC counts were determined using an automated hematology analyzer (Sysmex Corporation, Kobe, Japan). Serum CRP was measured by latex immunoassay (Hitachi, Tokyo, Japan), and ESR-1h was determined by the Westergren method [16]. For presepsin measurement, blood was collected in EDTA tubes and analyzed using a chemiluminescent enzyme immunoassay (PATHFAST^®^, Mitsubishi, Tokyo, Chemical, Japan) [17]. A cut-off value of 314 pg/mL was set according to the manufacturer’s instructions.

### 2.3. Statistical Analysis

We defined the sample size in this study as follows. Study 1 aimed to investigate the perioperative changes in inflammatory parameters and presepsin. Generally, when examining trends in a population, a sample size of 30 is considered sufficient, as the sample mean approaches the population mean, and the distribution of the sample mean approximates a normal distribution. Based on this rationale, the sample size was set to 31. In study 2, based on the results of study 1, we measured presepsin levels in cases suspected of postoperative infection. This study aimed to determine whether presepsin levels could show a statistically significant difference from baseline values before bacteria are detected in the blood. Given the low incidence of infection, approximately 0.2% [18], a minimum sample size of 4 was calculated, using a 95% confidence interval and a 5% margin of error [19]. Consequently, 5 subjects were selected.

The Shapiro–Wilk test was used to determine if the data (blood parameters) were normally distributed. The results showed that the distribution of WBC was considered to be normally distributed, while the distributions of presepsin, ESR-1h, and CRP were not considered to be normally distributed.

Categorical variables were shown as the number of each category, ages were shown as mean and standard deviations, and the continuous values of WBC, CRP, ESR-1h, and presepsin were each shown as a median and interquartile range [IQR] in accordance with their distributions.

For statistical analysis, the Kruskal–Wallis test was used to compare sequential changes in each variable, followed by the Sheffe comparison for each preoperative value. All data were analyzed using Bell Curve version 2.15 for the Excel program (Social Survey Research Information Co., Ltd., Shinjuku, Japan), and *p*-values < 0.05 were considered significant.

### 2.4. Result

**Figure 1:**
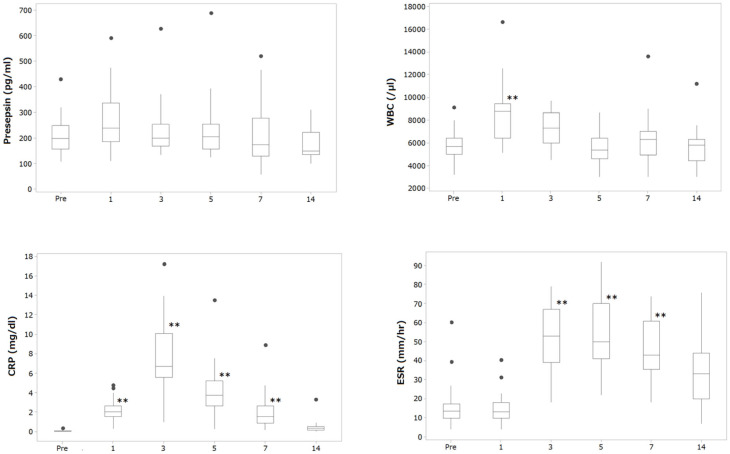
Sequential changes in each parameter before and after operation. Dot plot indicates an outlier. Significant difference from preoperative value is identified by post hoc Scheffe test, ** *p* < 0.01.

Table 3 shows the demographic characteristics in patients who underwent primary THA. The average age of the patients at the operation was 65.1 years. Twenty-four patients were female, and five were male. Figure 1 and Table 4 show the sequential changes of each parameter before and after the operation.

The Kruskal–Wallis test revealed statistically significant differences in values during the monitoring time in every parameter: presepsin (*p* < 0.01), WBC (*p* < 0.001), CRP (*p* < 0.001), and ESR-1h (*p* < 0.001). However, the post hoc test showed that significant changes in values from the baseline were observed in WBC at day 1 (*p* < 0.001), in CRP during days 1 to 7 (*p* < 0.001), and in ESR-1h during days 3 to 7 (*p* < 0.001) after surgery, but presepsin levels after surgery were not significantly different from the baseline during the monitoring period.

### 2.5. Study 2

We retrospectively evaluated 10 patients with suspected hip PJI at our hospital (May 2015–September 2018) using the 2018 MSIS diagnostic criteria [20].

During the diagnostic procedure, peripheral blood tests and peripheral blood cultures were first carried out for suspected PJI cases. If the blood tests showed an increased CRP and/or ESR-1h, aspiration to the affected joint was performed. Then, we decided the treatment strategy for PJI according to the guidelines recommended by the Infectious Diseases Society of America [21].

**Figure 2:**
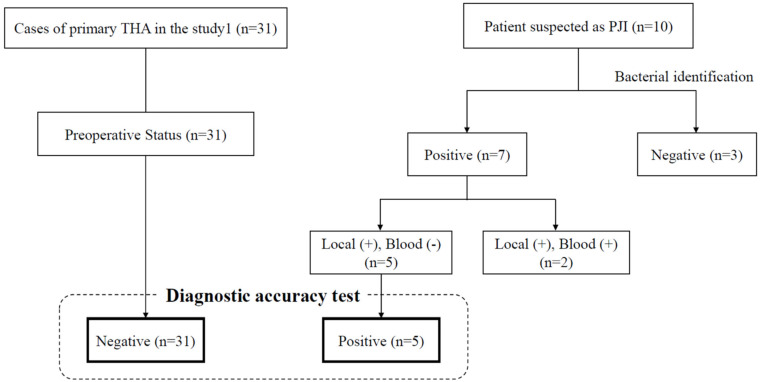
Flowchart on the accuracy test for PJI diagnosis. Abbreviations, THA; Total hip arthroplasty, PJI; Periprosthetic joint infection, +; positive bacterial culture, −; negative bacterial culture.

Of the PJIs, bacterial infections were identified from local specimens in seven cases and from peripheral blood samples in two cases. Because bacteremia affects the serum presepsin level, the latter two cases were excluded for the accuracy test. Finally, serum presepsin, WBC, CRP, and ESR-1h in 5 PJI cases (4 cases for ESR-1h) without bacteremia and 31 cases before primary THA were used for the accuracy test (Figure 2).

### 2.6. Measurement Protocol

Presepsin, WBC, CRP, and ESR-1h were examined on the same days that aspiration or surgical procedures on affected joints were performed for each patient. All of the blood samples were collected before antibiotic agents were systemically administered. A peripheral blood sample was collected by vein puncture, and each parameter was examined with the same procedure as was used for study 1.

### 2.7. Statistical Analysis

The discrimination of PJI was tested using the receiver operating characteristic (ROC) curve and area under the curve (AUC) for each parameter. AUC is an effective way to verify the diagnostic accuracy of a test [22,23]. In general, an AUC of 0.5 suggests no discrimination, 0.7 to 0.8 is considered acceptable, 0.8 to 0.9 is considered excellent, and more than 0.9 is considered outstanding [24]. Data were analyzed using BellCurve for Excel, and *p*-values < 0.05 were considered significantly different to the null hypothesis, which is defined as H0: AUC = 0.5.

### 2.8. Result

Table 5 shows each blood parameter in seven patients diagnosed with PJI. Case 7 demonstrated a markedly elevated presepsin level of 1700 pg/mL, a notable outlier representing a fivefold increase above the mean values observed in other cases, coinciding with severe septic inflammation. In the five-case series, each parameter showed ranges of 170–271 pg/mL for presepsin, 4800–15,600 /μL for WBC, 0.44–5.36 mg/dL for CRP, and 12–56 mm/h in the ESR-1h.

The ROC curve analysis revealed the following AUC values [95% CI]: presepsin 0.51 [0.24, 0.77], WBC 0.65 [0.37, 0.94], CRP 1.0 [1.0, 1.0], and ESR-1h 0.83 [0.57, 1.09]. The AUCs of CRP and ESR-1h were considered to discriminate PJI with cut-off values of 0.44 (mg/dL) and 33 (mm/h), respectively; however, those of presepsin and WBC did not demonstrate diagnostic value. Table 6 shows the sensitivity and specificity of presepsin for PJI diagnosis.

## 3. Discussion

In study 1, we assessed the normative perioperative changes in plasmatic presepsin, compared with WBC, CRP, and ESR-1h, in patients undergoing primary THA. We found that levels of presepsin showed no significant differences from the baseline value during the 2 weeks after surgery, despite significant changes in the levels of other inflammatory parameters. Despite the use of bone cement, which can potentially induce a stronger local surgical stress, presepsin levels remained unaffected. This highlights the robustness of presepsin against both surgical trauma and cement-related stress responses. In study 2, although we found an abrupt increase in presepsin in a patient with PJI and septic inflammation, the ROC analysis of presepsin failed to detect the presence of local infection in patients without bacteremia. Case 7 represents a notable exception, with presepsin levels reaching 1700 pg/mL, suggesting that once PJI progresses to systemic inflammatory response, presepsin may become markedly elevated. This exceptional case highlights presepsin’s potential utility in monitoring disease progression or detecting advanced infections where bacterial translocation from the joint to the bloodstream has occurred. However, for the crucial early diagnosis of contained PJI—the clinical scenario where biomarkers are most needed—presepsin appears to lack sensitivity. The results indicate that serum presepsin would not be useful for PJI’s diagnosis.

In the past few decades, many studies have attempted to seek a feasible biomarker for the diagnosis of PJI, because PJI has been a topic of considerable interest [25,26]. Inflammatory biomarkers, including cytokines, WBC, CRP, ESR, interleukin-6 (Il-6), procalcitonin, D-dimer, tumor necrosis factor-α (TNF-α), and intercellular adhesion molecule-1, have been investigated as potential markers for PJI’s diagnosis. To date, CRP and ESR remain the first-line options [2]. As indicated by this recommendation, only CRP and ESR were found to be potential markers for diagnosing PJI in this study.

Presepsin is a relatively new inflammatory marker developed at Iwate Medical University and expected to have broad clinical applications. Yaegashi et al. reported that presepsin values of sepsis patients were found to be significantly higher than in SIRS patients who did not have an infection [5]. Because one production mechanism of presepsin is related to the phagocytosis of bacteria, the biological characteristic of presepsin is different from other inflammatory markers.

An essential aspect of inflammatory biomarkers for indicating PJI is to have potential cut-off points between noninfectious and infectious status. Because a previous study showed that “normal control” values of presepsin obtained from 128 healthy Japanese subjects were variable (e.g., 294.2 ± 121.4 pg/mL), we needed to confirm the variability in serum presepsin levels from operative invasion throughout perioperative period. In study 1, we found that serum presepsin was seldom affected by surgical invasion, unlike other inflammatory markers. A previous study reported that presepsin levels were significantly increased at 2–4 days after surgery in patients who underwent THA; however, presepsin levels did not significantly differ from baseline until 4 days after surgery in patients who underwent total knee arthroplasty [14]. Their results indicated that serum presepsin levels were not strongly affected by surgical invasion. Giavarina et al. reported that the upper reference limit for presepsin obtained from individuals without inflammatory conditions was 184 pg/mL [9]. Even when applying this reference value as a cut-off point in our sample, presepsin demonstrated an extremely poor diagnostic performance, with a sensitivity and specificity of only 40% and 39%, respectively. These values are substantially below clinically acceptable thresholds, clearly indicating that serum presepsin may not be suitable for the early diagnosis of localized PJI, likely due to its dependence on systemic immune activation such as bacteremia. Its potential role may lie in assessing systemic infection’s progression rather than early localized joint infection.

Marazzi et al. have recently reported that serum presepsin could be considered a useful tool for the diagnosis and clinical monitoring of PJI [27]. They reported that serum presepsin levels statistically differed between infected patients and noninfected patients. However, whether the subjects in their study had bacteremia was unclear; moreover, a cut-off value was not reported. Given that the presepsin level was associated with septic severity [6,15,28], it can be inferred that some of the infected subjects had bacteremia, which affected the serum presepsin level. In the current study, we confirmed that serum presepsin in one patient (case 7) with septic inflammation as well as PJI was abruptly increased. However, the serum presepsin level of case 6 with bacteremia was not different from the baseline range in study 1. Therefore, we speculate that serum presepsin levels increase when an immunological reaction to a pathogenetic factor, such as phagocytosis to bacteria, occurs in the peripheral blood. The results of the present study also indicate that serum presepsin, as with procalcitonin [29], is not a useful biomarker for the diagnosis of PJI unless systematic bacterial infections are detected. Both biomarkers share a critical limitation: they primarily respond to systemic rather than localized infections. While they may effectively indicate the presence of bacteremia or systemic inflammatory response syndrome, neither appears sufficiently sensitive to detect the contained local inflammatory processes typical of early-stage PJI. This common limitation significantly restricts their clinical utility in routine PJI screening, where early detection of localized infection before systemic spread is paramount.

This study has several limitations. The first concern is the small sample size. Because the prevalence of PJI is approximately 1% for overall primary THAs [30], study 2 consisted of only five cases. Given the rarity of PJI, we applied epidemiologically relevant formulas; however, a power analysis for our hypothesis was not conducted. Consequently, the wide confidence interval (0.24–0.77) observed for the presepsin AUC demonstrates substantial statistical uncertainty in our analysis. Therefore, while this study is exploratory, no indications of localized PJI were observed.

Second, this study defined the preoperative status in each case as the control, although they had no prostheses in their bodies. A prosthesis in the body might affect the values of each parameter.

Third, our analysis did not stratify patients by important clinical variables such as acute versus chronic PJI, specific pathogen types, or the time course of infection. These factors potentially confound our findings, as different PJI presentations may exhibit varying inflammatory responses and consequently different presepsin profiles. This stratification would require a substantially larger cohort than was available for this study.

Additionally, our investigation lacks comparison with other emerging PJI-related biomarkers, such as α-defensin or interleukin-6 (IL-6), which have shown promise in recent literature. This limitation restricts the horizontal clinical value of our findings, as clinicians cannot determine how presepsin performs relative to these alternative biomarkers in the same patient population. Future studies should consider a comprehensive panel approach, comparing multiple biomarkers simultaneously to establish their relative diagnostic utility in PJI’s detection.

Fourth, we did not evaluate the comorbidities of the subjects, which may be associated with presepsin. Discrimination between septic and aseptic conditions after THA in a large multi-institutional sample is necessary.

## 4. Conclusions

In conclusion, while serum presepsin levels remained stable after THA and did not reliably discriminate localized PJI in this series, these findings are based on a small sample and should be interpreted cautiously. Further large-scale studies are needed to clarify its role, particularly in comparison with other emerging biomarkers such as IL-6 and α-defensin.

## Figures and Tables

**Table 1:** Patient background (no hip infection) and consent confirmation.

Sex	Age	Consent Obtained
F	62	consent
F	75	consent
F	61	consent
F	79	consent
F	68	consent
F	83	consent
F	65	consent
F	65	consent
F	67	consent
F	66	consent
F	61	consent
F	53	consent
F	74	consent
F	68	consent
F	75	consent
F	76	consent
F	76	consent
F	65	consent
F	53	consent
F	53	consent
F	68	consent
F	74	consent
F	68	consent
F	53	consent
F	64	consent
M	72	consent
M	52	consent
M	66	consent
M	63	consent
M	55	consent
M	46	consent

**Table 2:** Patient background (hip infection) and consent confirmation.

F	67	consent
M	74	consent
M	69	consent
M	56	consent
M	74	consent
F	69	consent
F	81	consent
M	69	consent
M	48	consent
F	73	consent

**Table 3:** Demographic parameters.

Parameter	
Number of subjects	29
Gender (M/F)	5/24
Number of side (L/R)	7/24
Age at surgery	65.4 (9.1)
Disease type	
Osteoarthritis	26
Ischemic optic neuropathy	1
Traumatic hip arthrosis	3
Rapidly destructive coxarthropathy	1

**Table 4:** Values of inflammatory markers before and after surgery.

Serum Biomarkers		Before Surgery	After Surgery				
			Day 1	Day 3	Day 5	Day 7	Day 14
Presepsin (pg/mL)	Mean (SD)	207.4 (67.4)	269.9 (109.9)	222.2 (93.8)	226.2 (109.6)	214.6 (113.3)	178.6 (59.2)
	IQR	162.0, 249.5	195.0, 323.3	168.0, 244.0	159.0, 244.8	130.5, 271.0	137.0, 221.0
WBC (/μL)	Mean (SD)	5783.9 (1435.8)	8446.7 (2387.6)	7217.4 (1479.1)	5616.1 (1360.9)	6240.0 (2014.7)	5603.4 (1547.5)
	IQR	5100, 6350	6625, 9400	6100, 8250	4750, 6350	5050, 7000	4600, 6300
CRP (mg/dL)	Mean (SD)	0.10 (0.09)	2.10 (1.11)	7.46 (3.75)	4.04 (2.51)	2.06 (1.72)	0.49 (0.59)
	IQR	0.04, 0.13	1.64, 2.56	5.57, 9.36	2.70, 5.13	0.90, 2.56	0.19, 0.58
ESR (mm/h)	Mean (SD)	15.7 (11.1)	14.4 (7.4)	50.1 (17.4)	54.6 (18.9)	46.2 (16.2)	31.6 (15.9)
	IQR	10.0, 17.0	10.3, 17.5	39.5, 66.5	42.5, 67.5	36.3, 60.0	20.0, 43.3

SD: standard deviation. IQR: interquartile range.

**Table 5:** A case series of hip infection.

Case No	Age	Sex	Blood Test				Bacterial Identification		Sinus Tract
			Presepsin (pg/mL)	WBC (/μL)	CRP (mg/dL)	ESR (mm/h)	Local	Blood	
1	67	F	170	4800	0.96	56	*Enterococcus. faecalis*	No growth	−
2	74	M	170	7300	1.49	N/A ^#^	*Staphylococcus caprae*	No growth	−
3	69	M	179	6000	1.06	12	*MRCNS* (*Staphylococcus caprae*)	No growth	−
4	56	M	271	15,600	5.36	56	*MRSA*	No growth	+
5	74	M	246	5900	0.44	48	*MRCNS* (*Staphylococcus epidermidis*)	No growth	+
6	48	M	244	7100	2.56	54	*MRSA*	*MRCNS* (*Staphylococcus haemolyticus*)	*+*
7	73	F	1700	12,200	4.97	99	*MRCNS* (*Staphylococcus epidermidis*)	*MRCNS* (*Staphylococcus epidermidis*)	−

^#^ ESR of case 2 was not collected. Abbreviations, MRSA: Methicillin-resistant Staphylococcus aureus MRCNS: Methicillin-resistant coagulase negative staphylococci, +; positive, −; negative.

**Table 6:** Sensitivity and specificity of serum presepsin for PJI diagnosis.

Presepsin Level	Sensitivity	Specificity
170	100%	29%
174	60%	29%
177	60%	32%
179	60%	35%
184	40%	39%
190	40%	45%
199	40%	48%
201	40%	55%
215	40%	58%
219	40%	61%
235	40%	65%
238	40%	68%
246	40%	71%
249	20%	71%
250	20%	74%
260	20%	77%
268	20%	81%
271	20%	87%
272	0%	87%
278	0%	90%
320	0%	94%
428	0%	97%
429	0%	100%

## Data Availability

The original contributions presented in this study are included in the article. Further inquiries can be directed to the corresponding authors.
